# Phylogeography of the Central American lancehead *Bothrops asper* (SERPENTES: VIPERIDAE)

**DOI:** 10.1371/journal.pone.0187969

**Published:** 2017-11-27

**Authors:** Mónica Saldarriaga-Córdoba, Christopher L. Parkinson, Juan M. Daza, Wolfgang Wüster, Mahmood Sasa

**Affiliations:** 1 Centro de Investigación en Recursos Naturales y Sustentabilidad, Universidad Bernardo O´Higgins, Santiago, Chile; 2 Department of Biology, University of Central Florida, Orlando, Florida, United States of America; 3 Grupo Herpetológico de Antioquia, Instituto de Biología, Universidad de Antioquia, Medellín, Colombia; 4 School of Biological Sciences, Bangor University, Bangor, United states of Kingdom; 5 Instituto Clodomiro Picado, Universidad de Costa Rica, San José, Costa Rica; 6 Organization for Tropical Studies, San José, Costa Rica; 7 Escuela de Biología, Universidad de Costa Rica, San José, Costa Rica; National Cheng Kung University, TAIWAN

## Abstract

The uplift and final connection of the Central American land bridge is considered the major event that allowed biotic exchange between vertebrate lineages of northern and southern origin in the New World. However, given the complex tectonics that shaped Middle America, there is still substantial controversy over details of this geographical reconnection, and its role in determining biogeographic patterns in the region. Here, we examine the phylogeography of *Bothrops asper*, a widely distributed pitviper in Middle America and northwestern South America, in an attempt to evaluate how the final Isthmian uplift and other biogeographical boundaries in the region influenced genealogical lineage divergence in this species. We examined sequence data from two mitochondrial genes (*MT-CYB* and *MT-ND4*) from 111 specimens of *B*. *asper*, representing 70 localities throughout the species’ distribution. We reconstructed phylogeographic patterns using maximum likelihood and Bayesian methods and estimated divergence time using the Bayesian relaxed clock method. Within the nominal species, an early split led to two divergent lineages of *B*. *asper*: one includes five phylogroups distributed in Caribbean Middle America and southwestern Ecuador, and the other comprises five other groups scattered in the Pacific slope of Isthmian Central America and northwestern South America. Our results provide evidence of a complex transition that involves at least two dispersal events into Middle America during the final closure of the Isthmus.

## Introduction

Tropical Middle America, the region that extends from the Isthmus of Tehuantepec to the northwestern tip of South America, accounts for less than 0.7% of the Earth’s total land area, and yet, is one of the most biologically diverse territories on the planet [1–3]. The origin and maintenance of its tremendous diversity is attributed not only to Mesoamerica’s geographical location between two major continental masses but also to the intricate geological and climatological history that shaped the region since the late Cretaceous [4–6].

Among the myriad of events that resulted from these dynamics, perhaps the most significant was the rise and final connection of the Lower Central American Isthmus (LCA, but also known as the Isthmus of Panama). The uplift did not occur as a single incident but instead resulted from a series of geological and climatological events that started in the Oligocene and continued until the mid-Pliocene when the unbroken connection finally emerged [5–10, but see 11 for a different view]. This process allowed not only the evolution and redistribution of organisms in Mesoamerica [12, 13] but also was crucial in the exchange of species between the Northern and Southern continents, often referred as the Great American Biotic Exchange [14–18]. Despite its importance, there is still considerable debate about the timing of the final LCA connection, and whether pre-closure dispersal or vicariant events mediated the cladogenesis and biological diversification observed in the region [19, 20].

Several studies have shown the effectiveness of molecular phylogeographic approaches combined with robust estimations of divergence time to assess the role of putative geological events in the cladogenesis of species inhabiting Middle America [20–22]. This has allowed scientists to address questions about how historical divergence occurred across landscapes, especially in regions where there is little consensus on historical processes, as is the case of Middle America.

Pitvipers have been used to elucidate fine-scale historical biogeographical patterns, [19, 23–25] because most species have lower vagility than other vertebrates, which make them more prone to genetic isolation via vicariance. Moreover, the intrageneric relationships of pitvipers in Middle America are well known [26–30], and it seems that Mesoamerican pitviper lineages exhibit coincident temporal patterns of divergence that match major geological events that shaped the region. For instance, the tropical rattlesnake *Crotalus durissus* (*sensu lato*) has a northern origin [31], but its phylogeographic pattern is consistent with a gradual range expansion south that corresponded to the final uplift of the LCA isthmus, followed by a rapid dispersal into South America [32].

The effect of the LCA uplift and final closure on tropical snake lineages of South American origin is far less understood, although it is believed that most dispersed north only after the uplift of the Isthmus [29]. To assess the effect of the LCA uplift on South American taxa, we focus on the Central American lancehead pitviper, *Bothrops asper*. This species has close affinities with members of the *B*. *atrox* complex [sensu 27] a group mainly distributed east of the Andes [33], and is thus nested deeply within the genus *Bothrops* that is otherwise confined to South America [26, 27, 34]. However, *B*. *asper* extends its distribution from Colombia and northwestern Peru in South America to lowland Mexico and Central America. Given these affinities and its current distribution well into Middle America, it appears that *Bothrops asper* expanded its range rapidly northward once the LCA isthmus was finalized [34]. Thus, we predict that *B*. *asper* populations in Middle America will be more recent and exhibit lower divergence than those in South America.

To test this, we assess the phylogeographic pattern of *B*. *asper* using mtDNA markers across the species range and evaluate the time and mode in which the species colonized Mesoamerica.

## Materials and methods

### Specimens and laboratory methods

**Fig 1:**
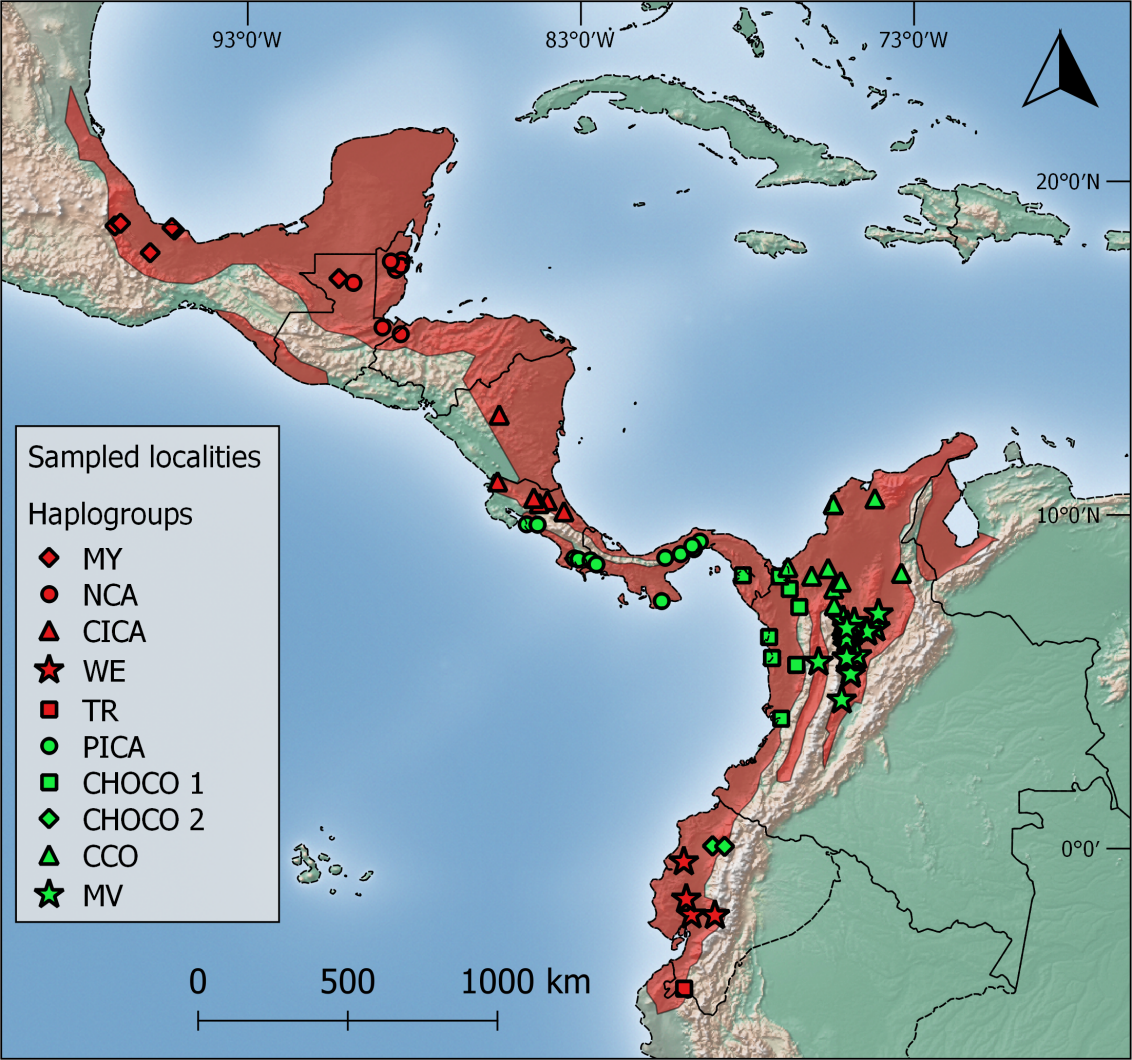
Distribution of *Bothrops asper* (red contour) in Middle and South America adapted from [33]. Question marks in northwestern Venezuela and Peru indicate that part of the distribution which needs confirmation. Symbols represent localities of specimens included in analyses, showing their phylogeographical affinities: MY (Mexico-Yucatan), NCA (Caribbean Nuclear Central America), CICA (Caribbean Isthmian Central America), PICA (Pacific Isthmian Central America), WE (West Ecuador), TR (Tumbes region), CHOCO 1 (Darien-Colombian Chocó), CHOCO 2 (Ecuadorian Chocó), CCO (Caribbean Colombia), MV (Magdalena Valley). See text for elaboration.

*Bothrops asper* individuals were collected from 70 localities throughout the species distribution (Fig 1 and S1 Table). Genomic DNA was extracted mostly from blood or shed skin following the procedure described in [35]. The use of biological material for this study was approved by the Research Committee of Instituto Clodomiro Picado (session No 06–2013) and by the Institutional Committee for Care and Use of Animals (CICUA) from Universidad de Costa Rica.

The *MT-ND4* and *MT-CYB* regions were amplified using the primer pairs described in [36, 37] respectively. For both genes, PCR reactions were set up to a final volume of 25 μl, using 2.0 μl genomic DNA, 0.4 μl of each primer (0.16 μM), 2.5 μl of 10 X PCR buffer (1X), 0.25 μl total dNTPs (100 μM), 1.0 μl of MgCl_2_ (2 mM), 0.2 μl of Taq polymerase (1 U), and 18.6 μl H_2_O). Typical amplification conditions involved initial denaturation at 94°C for 5 min, followed by 38 cycles of 94°C for 40 s, 54°C of annealing for 40 s, then 72°C for 1 min, followed by a final extension step of 72°C for 5 min. The amplified product was sequenced using the same primers by Macrogen (Seoul, S. Korea– http://dna.macrogen.com).

### Alignment and data exploration

DNA sequences were edited using BioEdit version 7.0 [38], and then the alignment was verified by eye in GeneDoc [39]. Since we use coding genes in our analyses, all nucleotide sequences were translated into amino acids to evaluate the reading frame and ensure the absence of premature stop codons or other nonsense mutation [40]. We deposited novel sequences were deposited in GenBank, and the final nucleotide alignment matrix is available upon request. We calculated genetic distances (p-distance) from aligned sequences using Mega version 7 [41].

### Phylogenetic analysis and divergence time estimation

For phylogenetic analyses we incorporate representatives of the following genera (# species) as outgroups: *Lachesis* (2), *Ophryacus* (2), *Agkistrodon* (2), *Sistrurus* (1), *Crotalus* (2), *Bothriocophias* (3), *Rhinocerophis* (4), *Bothropoides* (11), *Bothriopsis* (3), and *Bothrops* (12) (S1 Table).

We treated gaps in the alignment as missing data. We concatenated the different gene sequences (*MT-ND4* and *MT-CYB*) for each specimen and analyzed the data jointly. Data for each gene was partitioned following [29], and Mega version 7 [41] was used to estimate the best-fitting models of nucleotide evolution for each partition independently. We select the best-fitting models according to Bayesian Information Criterion (BIC) [42].

We performed phylogenetic analyses of concatenated genes using maximum likelihood (ML), and Metropolis-Hastings coupled Markov chain Monte Carlo Bayesian methods (BMCMC). ML analyses were performed under different models of nucleotide evolution [43]. Initial tree(s) for the heuristic search were obtained automatically by applying Neighbor-Join and BioNJ algorithms to a matrix of pairwise distances estimated using the Maximum Composite Likelihood (MCL) approach and then selecting the topology with superior log-likelihood value. We used a discrete Gamma distribution to model evolutionary rate differences among sites. These analyses were conducted in MEGA7 [41]. We use non-parametric bootstrap (10,000 replicates) to evaluate branch support in the phylogenetic reconstruction [44].

The BMCMC estimate of the phylogeny was inferred using MrBayes version 3.0B4 [45]. We executed three parallel MCMC runs simultaneously, each run for 20 x 10^6^ generations with four Markov chains (one cold and three heated chains). Model parameters and the rate of evolution differ among each partition. We used Tracer 1.6 [46] for visualizing output parameters to ascertain stationarity and whether or not the duplicated runs had converged on the same mean likelihood. Runs appeared stationary before 10^6^ generations, and we conservatively excluded the first 2.0 x 10^6^ generations of each run as burn-in. All post-burn-in estimates (sampled every 1000 generations) were combined, and we summarized phylogeny and parameter estimates from this combined posterior distribution. Nodes were considered well supported if posterior probabilities > 0.95.

To evaluate phylogroup boundaries on our preferred phylogenetic tree, we performed Bayesian Poisson Tree Process (bPTP) implemented in http://www.exelixis-lab.org/software.html [47]. This method considers the number of substitutions between branching (speciation events) and assumes that each substitution has a small probability of generating speciation. Thus, if the number of changes is sufficiently large, the process follows a Poisson distribution. bPTP adds Bayesian support (BS) values to delimited species on the input tree, with higher node support indicates that all descendants of this node are more likely to be from one species [47].

We also implemented the Bayesian phylogeography approach (BPA, [48–49]) to evaluate the putative area of origin of *B*. *asper*-*B*. *atrox* lineages. BPA takes into account space-time domains when analyzing the evolutionary process, and uses localities as discrete states to allow inferences about the location of ancestral lineages. In order to reconstruct the ancestral state, we re-ran our Bayesian analysis without outgroups, using BEAST v.2.4.7 [50]. Three individual runs were performed for 10 x 10^6^ generations with a sampling frequency of 10,000; under the same nucleotide substitution models used in our ML phylogenetic analysis (see Results). We apply the following parameters as priors: coalescence: constant size speciation process; clock rate: set at 0.005; and strict molecular clock. For each individual included in the analysis, we designate their country of origin as the state of locality. We are aware that this designation is broad and artificial, but we consider that it allows us to locate in present-day geography the spaces occupied by the ancestral lineages.

For all analyses performed on BEAST, each run was analyzed in Tracer [46] to confirm that effective sample sizes (ESS) were sufficient for all parameters (posterior ESS values > 300). LogCombiner® and TreeAnnotator® (both available in the BEAST package) were used to infer the ultrametric tree after discarding 10% of the samples from each run.

We estimated divergence times using the Bayesian relaxed clock method with uncorrelated lognormal rates among branches across the *B*. *asper* phylogeny, assuming a birth-death process for the speciation model implemented in BEAST v.2 [50]. This method incorporates heterogeneity rates based on Bayesian inference and allows the simultaneous use of different evolutionary parameters for each dataset [51, 52].

Posterior distributions of parameters were approximated using three independent MCMC analyses of 20 x 10^6^ generations each, with samples retained every 1,000 generations. Samples from the two runs, which yielded similar results, were combined and convergence of the chains was checked using the program Tracer 1.6 [46].

We used three calibration points: (1) the minimum age of *Sistrurus* estimated from the fossil evidence of the most recent ancestor (TMRCA) of *Sistrurus + Crotalus* [53]. Using a log-normal prior with zero offset (hard upper bound) of 8 million years ago (Mya), a mean of 0.01, and standard deviation (SD) of 0.76, this estimation produced a median age centered at 9 Mya and a 95% prior credible interval (PCI) extending to 11.5 Mya [54] (2) The basal divergence within the crown *Agkistrodon* clade (*A*. *piscivorous*-*A*. *contortrix* divergence) in the Late Miocene [54]. Using a log-normal prior with zero offset of 6 Mya, a mean of 0.01, and SD of 0.42, this estimation produced a median age of 7 Mya and a 95% PCI extending to 8 Mya [54]. Although these are very narrow constraints based on a patchy fossil record, true divergence dates will probably be older than the oldest known fossil, thus making these lognormal distributions with hard lower bounds a conservative assumption [25, 55]. (3) The estimated age of divergence between *C*. *ruber* and *C*. *atrox* according to [56] due to the Pliocene marine incursion of the Sea of Cortés [57], using a lognormal mean of 1.1 and SD of 0.37, no zero offset. This produced a median age centered at 3 Mya and a 95% PCI extending to 5.5 Mya that coincides with time estimations for the development of Sea of Cortés [57]. There is still some debate on the time and level of isolation of the California peninsula during those marine incursions [58], so we perform our analyses including and excluding this calibration point. No differences in the estimation of divergence time retrieved by both approaches were noticed.

### Testing for range expansion

We analyze the distribution of pairwise nucleotide differences (mismatch distribution) to infer the existence of demographic growth, following [59] method implemented in ARLEQUIN 3.5 [60]. Distribution curves were analyzed assuming constant and non-constant sizes for the observed empirical distribution. We use *rg* as a measure of "statistical raggedness" [61] and *R*^*2*^ statistic [62] to determine the correspondence between the observed and theoretical curves. Also, we employed Fu and Li [63], and Tajima ɵ statistics to detect possible changes in population sizes [62, 64].

## Results

### Sequence analysis

We generated new sequences for *B*. *asper* (*MT-CYB*: 101; *MT-ND4*: 90), *B*. *atrox* (*MT-CYB*: 5; *MT-ND4*: 1), *B*. *colombiensis* (*MT-CYB*: 9; *MT-ND4*: 9), *B*. *isabelae* (*MT-CYB*: 5; *MT-ND4*: 5) and *B*. *venezuelensis* (*MT-CYB*: 2; *MT-ND4*: 1). From 174 specimens, including 111 in the ingroup, we obtained a concatenated matrix of 1442 bp for the two genes (758bp for *MT-CYB* and 684bp for *MT-ND4*).

The alignment was unambiguous, and the inferred amino acid sequence contained no stop codons, which suggest a mitochondrial origin sequence rather than nuclear insertion. Including outgroups, we recover 545 polymorphic sites from all sequences, 387 of them are informative. For *B*. *asper*, we recover 296 polymorphic sites, 237 informative.

### Phylogenetic analysis and genetic distances

**Fig 2:**
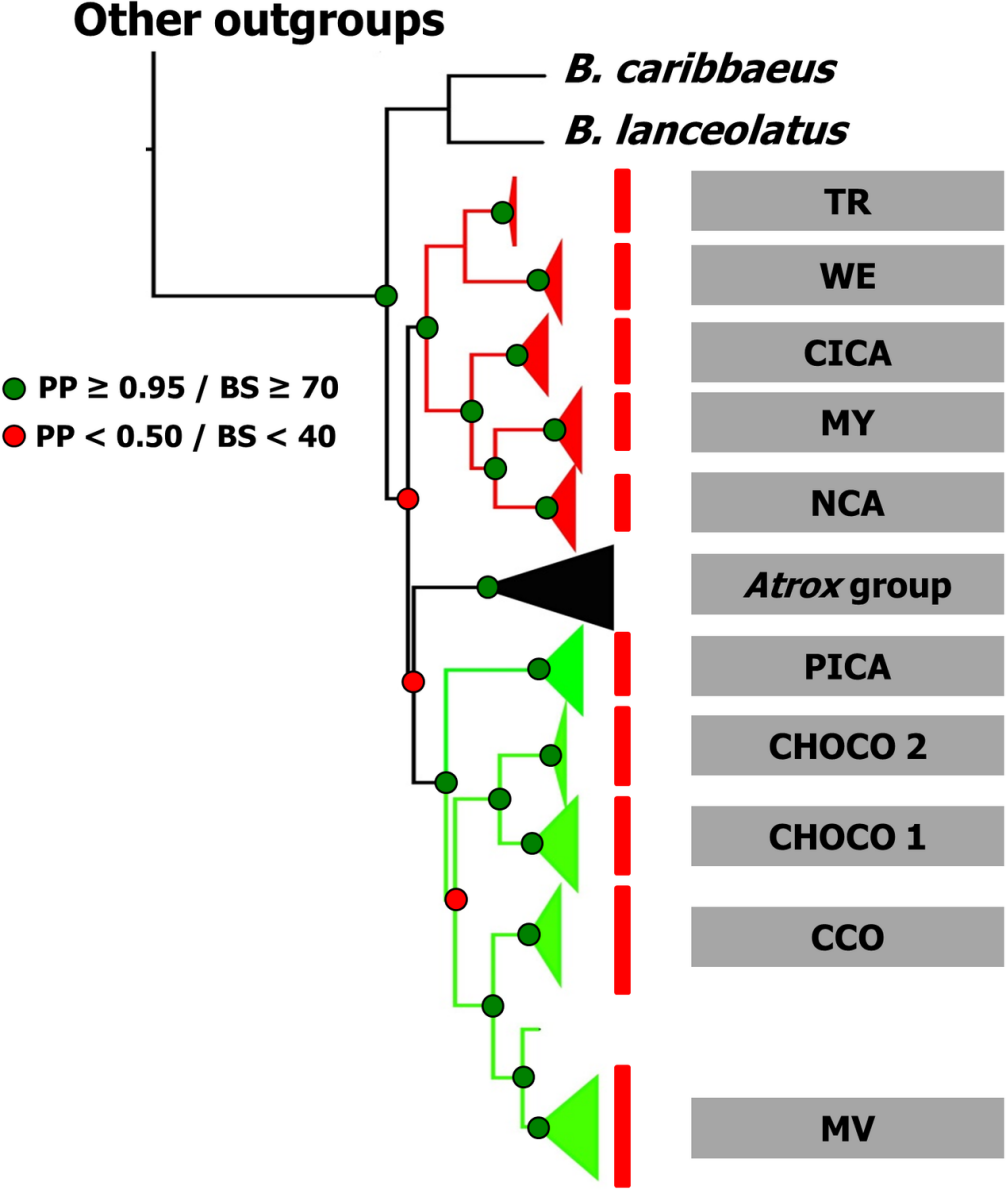
Bayesian phylogeny of relationships among members of *B*. *asper* from different physiographic regions (names as in Fig 1). Two distinct B. asper lineages are depicted in red (clade A) and green (clade B) branches. Red bars indicate phylogroups supported by the bPTP analysis. Support for each node is shown as posterior probability (PP, Bayesian inference) or Bootstrap value (BS, Maximum likelihood).

Selected models of nucleotide evolution differ among all partitions, the model chosen was GTR+Г for the first and third codon position of *MT-CYB* and all *MT-ND4* partitions, and HKY+ I for the second *MT-CYB* position. Under ML, the tree with the highest log likelihood (-9078.04) is congruent with the phylogenetic relationships recovered under the BMCMC method, with only minor differences among the outgroups. Thus, we only show the results of the Bayesian analysis here (Fig 2).

Our analysis recovered a partial phylogeny of *Bothrops* that is consistent with previous reconstructions of lancehead phylogenies based on morphology and/or mtDNA [30, 34, 65–68].

**Fig 3:**
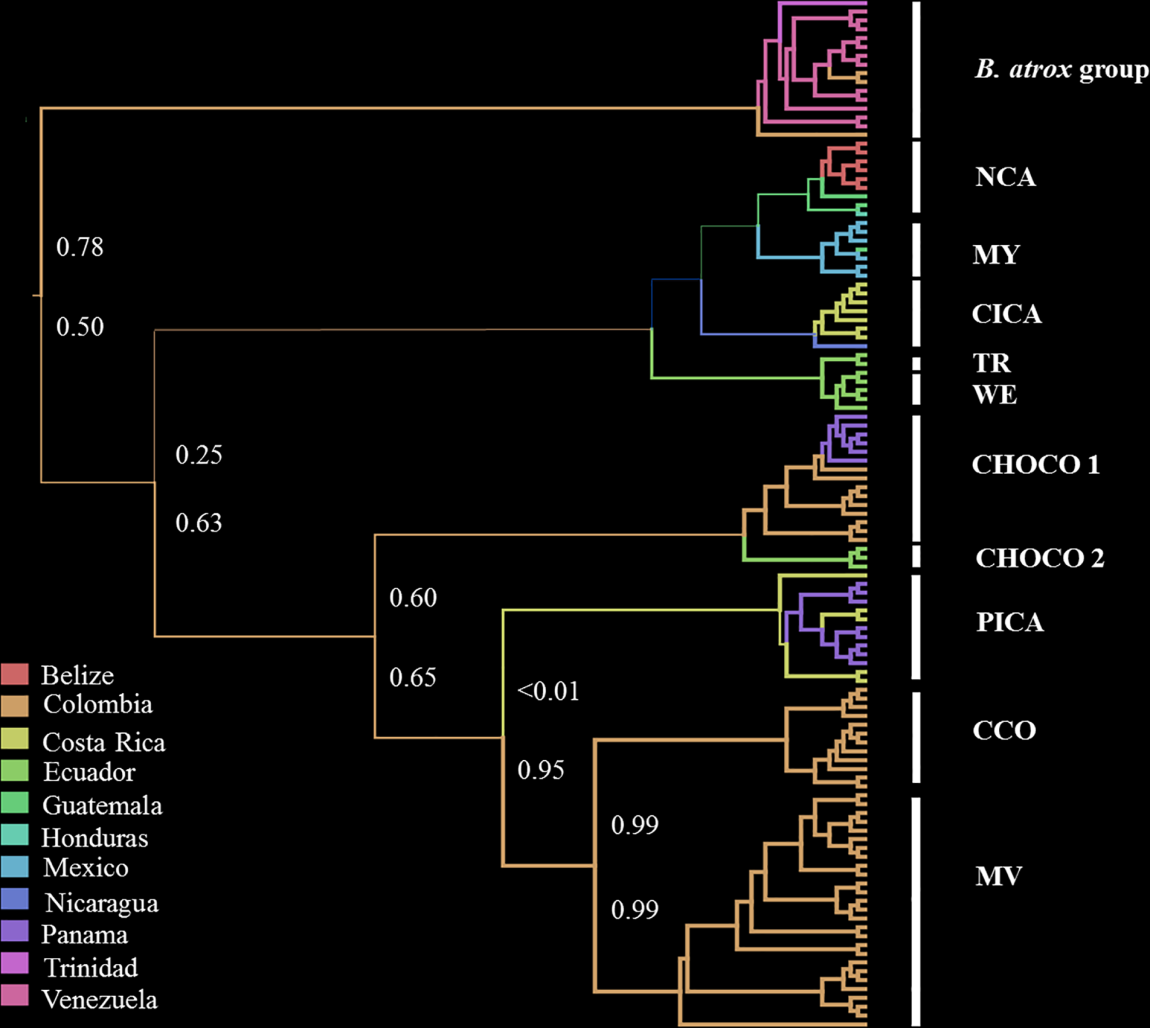
Maximum clade credibility phylogeny for *Bothrops asper*, retrieved from mtDNA sequences. Branches are colored according to the most probable “location state” of their descendant nodes. Values in branches indicate the location set probability of the ancestral state, in this case, the probability that the origin of the branch occurred in the region that is now Colombia. Bayesian support for clades as in Fig 2.

**Fig 4:**
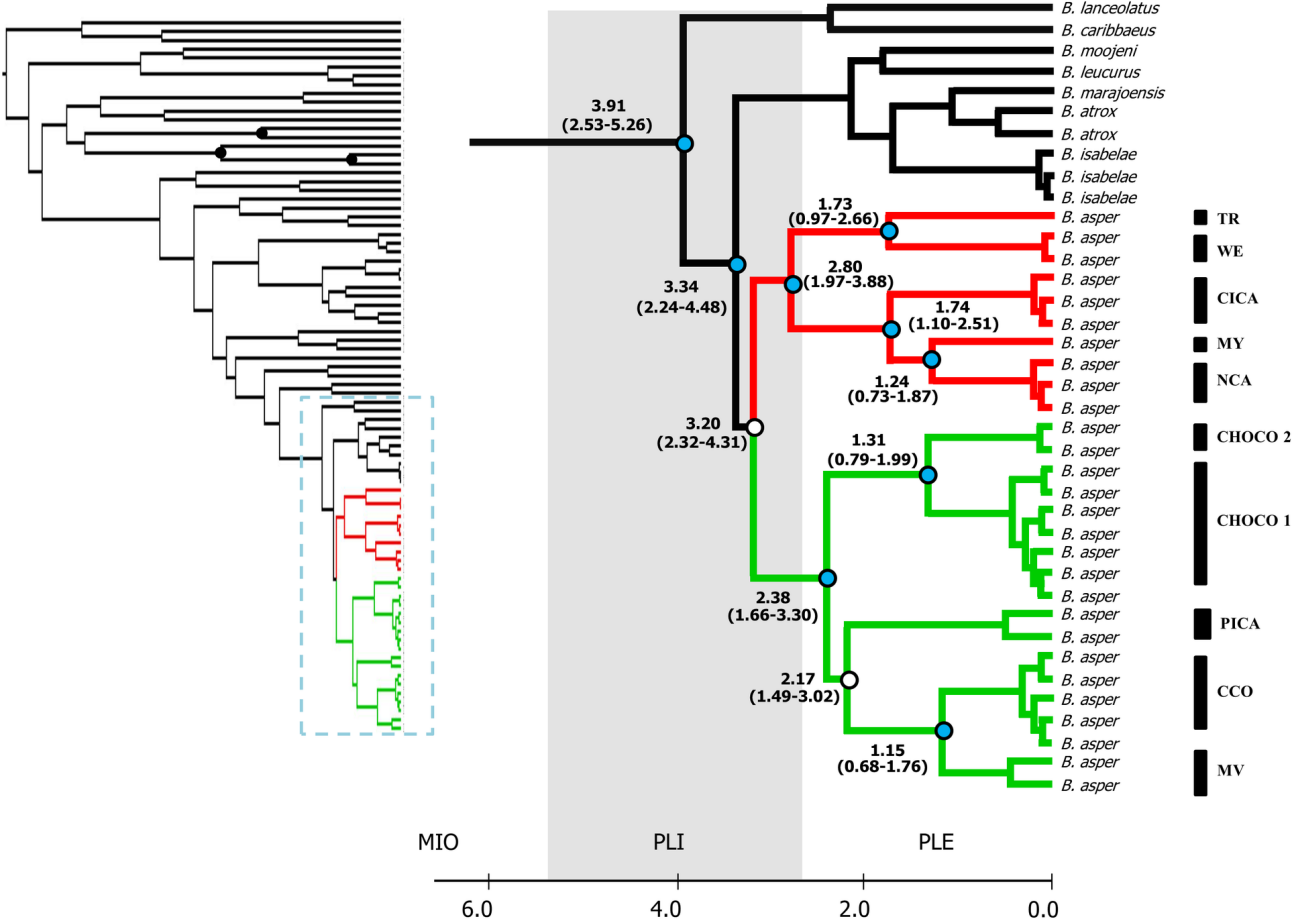
Bayesian estimates of divergence time (Mya) for the lancehead phylogeny. Left: Overall tree showing the calibration points (black dots) for time divergence estimations. Right: *B*. *asper* phylogeny showing the mean and 95% confidence intervals (in parenthesis) for divergence time estimates at each node. Clades A and B are depicted as in Fig 2. Grey bar indicates the extension of the Pliocene. Well supported nodes (PP > 0.95) for divergence estimations are shown in light blue, whereas weakly supported nodes are shown in white. *B*. *asper* phylogroup names as in Fig 1.

We recovered a deep split of *B*. *asper* into two well-supported lineages (clades A and B, Fig 2), differing by average p-distances of 5.7% (*MT-CYB)* and 4.3% (*MT-ND4*), and paraphyly of *B*. *asper* concerning the *B*. *atrox* group. However, the node placing the *atrox* group as sister to *B*. *asper* clade B (Fig 2) is weakly supported, and our analyses using BEAST recovered *B*. *asper* as monophyletic (see Figs 3 and 4).

Clade A includes *B*. *asper* specimens from the Caribbean coast of Middle America and southwestern Ecuador. In this clade, five well supported (PP > 0.9) groups were retrieved in our bPTP analysis (Fig 2): (1) specimens from localities along the Gulf of Mexico coast and Yucatán Peninsula, including Petén (MY); (2) Nuclear Central America (NCA), including specimens from the Caribbean slopes of Honduras, Guatemala, and Belize; (3) Caribbean Isthmian Central America (CICA), from the Caribbean lowlands of Nicaragua and Costa Rica; (4) Western Ecuador (WE), from Guayas, Manabí, Los Ríos and Chimborazo provinces in Ecuador; (5) Tumbes biogeographic region (TR, that includes specimens from Loja in southern Ecuador). MY and NCA form a well-supported clade that is distributed within the same biogeographical unit (Veracruzan Province *sensu* Morrone [69]).

Clade B also includes five distinct *B*. *asper* groups (Fig 2): (1) Pacific Isthmian Central America (PICA) from Pacific localities of Costa Rica and Panama; (2) Northern Choco (CHOCO 1) from central and eastern Panama, and Pacific coast of Colombia; (3) Chocoan Ecuador (CHOCO 2), from Esmeralda and Pichincha, Ecuador; (4) Caribbean Colombia (CCO) localities from the Caribbean lowlands of Colombia; and (5) Rio Magdalena valley (MV), from localities along the upper Magdalena basin. CHOCO 1 and CHOCO 2 form a well-supported clade that is distributed within the same biogeographical unit (Choco-Darien Province [69]). The groups spread west of the Cordillera Occidental (CCO and MV) also form a supported clade (Fig 2) and are included in Morrone’s Magdalena Province [69].

Average uncorrected *p-*distances for *MT-ND4* were lower than those for *MT-CYB* in most groups (Table 1). The pairwise divergence between the *B*. *atrox* group and *B*. *asper* lineages ranged between 3.1% and 6.1% for *MT-CYB*, and 4.3% and 5.9% for *MT-ND4*. The separation between *B*. *asper* clades A and B ranged between 3.6% and 8.2% for *MT-CYB* and 3.3% and 5.1% for *MT-ND4*: the greatest pairwise divergence observed between individuals from MY and those from CCO in Colombia for (*MT-CYB p* distance = 8.2%, Table 1). Even in Costa Rica, the mean pairwise divergence between snakes from the Caribbean region (CICA) and those in the Pacific lowlands (PICA) was relatively high, 6.5% (*MT-CYB*) and 4.6% (*MT-ND4*), despite that these populations occur only a few kilometers apart. The lowest mean divergence was noticed between snakes distributed in the Magdalena Valley and those in the Caribbean region of Colombia (Table 1).

**Table 1:** Net divergences (uncorrected p-distances) for *MT-CYB* (below diagonal) and *MT-ND4* (above diagonal) within *B*. *asper* groups, and among them and related clades. For group names see text.

	Groups	WGMD	1	2	3	4	5	6	7	8	9	10	11	12
**1**	**Antillean Bothrops**	3.9\4.5		5.0	4.9	4.0	3.7	4.8	4.9	5.2	4.6	4.5	4.4	4.3
**2**	***B*. *atrox* group**	2.6\2.9	4.1		5.0	4.5	4.3	4.4	5.2	5.9	5.0	5.0	4.5	5.1
**3**	**MY**	0.1\0.2	5.3	6.0		3.4	2.0	4.4	4.8	5.1	4.5	4.6	4.7	4.7
**4**	**NCA**	0.0\01	4.5	5.7	2.7		1.3	3.7	3.6	4.9	3.5	3.7	3.7	3.8
**5**	**CICA**	0.1\0.2	4.6	4.6	4.0	3.9		3.4	3.8	4.6	3.3	3.3	3.4	3.4
**6**	**TR**	0.0\0.0	3.7	3.1	4.9	4.3	3.1		3.1	4.8	4.4	4.3	4.3	4.8
**7**	**WE**	0.0\0.1	4.5	5.0	6.0	4.3	3.9	2.6		4.8	4.4	4.8	4.3	4.4
**8**	**PICA**	0.4\0.6	5.4	5.4	7.5	7.5	6.5	5.9	6.1		4.0	4.3	3.8	3.9
**9**	**CHOCO1**	0.2\0.4	4.5	3.4	5.3	6.0	4.9	3.6	5.1	4.1		1.5	2.5	3.0
**10**	**CHOCO2**	0.3\0.2	4.7	3.9	6.7	6.6	5.1	4.0	4.9	3.0	2.4		2.9	3.4
**11**	**CCO**	0.6\0.3	6.3	6.1	8.2	7.4	6.2	5.7	5.7	4.8	4.8	5.4		2.4
**12**	**MV**	0.1\0.3	5.2	5.2	6.5	6.5	5.3	5.6	5.7	3.9	3.0	4.5	1.8	

WGMD, Within Group Mean Distance.

Within *B*. *asper* groups, we observed the highest divergence among individuals in PICA (mean p-distances = 0.4% and 0.6%, for *MT-CYB* and *MT-ND4* gene, respectively); whereas we recorded the lowest value within the MV group (mean p-distances = 0.1 and 0.3%, for both genes).

We observed valuable sample sizes for all parameters in all BEAST analyses and our estimation of convergence statistics in Tracer indicated that all analyses had converged (ESS > 384). The root state posterior probabilities for all locations range between <0.01 and 0.78, with Colombia receiving the highest probability (Fig 3). Thus, the ancestral branch that led to the *B*. *atrox* complex exhibited high probability for that locality (P > 0.78), although it is somewhat lower in the branch leading to *B*. *asper* (P > 0.5, Fig 3). Similarly, the probability that Colombia was the state at the root of Clade B is high (P = 0.63), despite the low probability observed in the branch leading to PICA. On the other hand, neither Colombia nor any other locality dominates the probabilities associated with the root of Clade A, and therefore the location state of its ancestor remains unclear.

### Divergence times

According to our Bayesian estimations of divergence time (Fig 4) the origin of the *B*. *asper-B*. *atrox* group occurred approximately 3.91 Mya (CI_95%_ = 2.53 to 5.26 Mya) when this clade diverged from the Antillean lanceheads (*B*. *caribbaeus* and *B*. *lanceolatus*). The divergence between the *B*. *atrox* species group and the *B*. *asper* lineages is estimated to have occurred soon after, about 3.34 (CI_95%_ = 2.44 to 4.48 Mya). The separation between *B*. *asper* clades A and B is dated at mid-Pliocene, approximately 3.20 Mya (CI_95%_ = 2.32 to 4.31 Mya). Within clade A, the Caribbean Middle American groups diverged from those in Southwestern Ecuador almost simultaneously, as the estimation time is 2.80 Mya (CI_95%_ = 1.97 to 3.88 Mya).

The divergence between sister groups (MY-NCA/CICA) and (WE/TR) occurred more recently approximately 1.74 Mya (CI_95%_ = 1.10 to 2.51 Mya), at the Pliocene-Pleistocene boundary (Fig 4).

Within clade B, lineages from the Choco biogeographic region diverged from all other groups about 2.38 Mya (CI_95%_ = 1.66 to 3.30 Mya) coinciding with the separation between PICA and the groups located east of the Colombian Andes (CCO and MV, Fig 3). Finally, the divergence estimates between MV and CCO and between the Choco-Ecuador and Choco Colombia-Panama groups occurred well into the Pleistocene (Fig 4).

### Testing for demographic expansion

A total of 41 and 47 unique haplotypes were recovered from MT-CYB and MT-ND4 sequences respectively. MY, CICA, and CHOCO exhibit a relatively higher number of distinct haplotypes (5, 7, and 9, respectively), whereas we noticed only four in MV, despite this last group has the most extensive sample size (Table 2).

**Table 2:** Mistmatch distribution statistics (*MT-CYB* and *MT-ND4*) for *B*. *asper* phylogroups. Group names as in Fig 1. No data available for TR and CHOCO 2 due to small sample sizes.

Groups	N *CYB/ND4*	Mismatch distribution	Rg	R^2^	D*	Tajima's D (θ_T_)
MY	7/4	Unimodal	0.33/0.25	0.14/0.27	0.23/0.59	-0.88/0.59
NCA	9/9	Unimodal	0.07/0.18	0.18/0.22	0.23/-1.68	0.20/-1.51
CICA	8/6	Unimodal	0.14/0.06*	0.12/0.25*	-0.72/-1.26	-0.70/-1.23
WE	5/5	Bimodal	0.68/0.05	0.40/0.27	0.23/-1.05	-0.97/-1.05
PICA	13/11	Bimodal	0.14/0.06	0.11/0.15	-0.55/0.49	-0.56/0.01
CHOCO1	16/11	Unimodal	0.02/0.04	0.13/0.09*	-0.59 /-1.71	-0.56/-1.44
CCO	12/11	Unimodal	0.04/0.09	0.11/0.14	-0.79/-0.44	-0.64/-0.80
MV	5/30	Unimodal	0.50/0.07	0.13/0.08	-3.32*/-3.46*	-2.09*/-2.07*

*significant values (P < 0.001).

N, sample size

Rg, Raggedness statistic [61]

R^2^, correlation between observed and expected curves [62]

D*, statistic to detect changes in population sizes [63].

Except for WE and PICA, all other studied groups showed unimodal patterns of mismatch distribution curves (Table 2). Also, most groups exhibited low values for the neutrality test statistics applied here (Table 2). These combined results are often interpreted as an indication of selective sweep or population expansion [63–64]. However, in our analysis only MV show significant values for the Fu and Li’s D* and Tajima’s ɵ, therefore supporting signs of recent demographic growth in that group (Table 2).

## Discussion

*Bothrops asper* exhibits robust genetic partitioning that accounts for at least ten distinct mitochondrial phylogroups, included in two separate lineages. The groups occupy different geographic regions and show private haplotypes, indicating a clear division among them. We found evidence of transition zones only in Peten (Guatemala) and in central Panama, but in both cases, contact occurs between sibling groups within a single *B*. *asper* lineage (MY/NCA and PICA/CHOCO1, respectively). Conversely, we did no observe mitochondrial admixture between *B*. *asper* lineages, nor even in Isthmian Central America where they converge. Additional sampling in this region, especially in western Panama, could help to establish the extent to which these lineages are introgressing and the precise geographical boundaries in this apparent admixture zone.

### Phylogeographic history

The observed molecular variation might, in part, result from the complicated geological and climatic history that shaped the distribution of the species during the Pliocene-Pleistocene, in addition to the species own ability to spread into the new empty niches that arose during that period.

As stated before, the sister relationship between *B*. *asper* and the *B*. *atrox* group has been established previously [27, 34, 65]; and a monophyletic *B*. *asper* was recovered in our BEAST estimations of divergence time and the putative ancestral distributions of our clades. Our estimates of divergence time suggest that the *B*. *asper-B*. *atrox* group ancestor was distributed in South America no older than 4.48 Mya (Fig 4). Also, BPA analysis resulted in Colombia as that the most probable 'state of locality' for the deep branches in the phylogeny of *B*. *asper* (Fig 3). We interpret these combined results as evidence that the diversification of *B*. *asper* occurred in the northwestern region of South America by the early Pliocene.

The allopatric distribution of *B*. *atrox* group and *B*. *asper* lineages on each side of the Eastern Andes Cordillera, suggests that the final uplift of this mountain range played a significant role in the cladogenesis of these lanceheads. Thus, this Andean Cordillera reached no more than 40% of its present elevation during much of the Neogene, but intense mountain building followed in the late Miocene and, especially in the Pliocene when elevations increased rapidly [70–71]. As a result, we hypothesize that a *B*. *asper* stock diverged to the west of the Eastern Andes, likely within the foothills along the Pacific coast of northern South America, as supported by the relatively high number of haplotypes observed in that region.

During that time, this ancient stock was further isolated by several marine incursions in northern South America, especially at the Magdalena Valley and the Maracaibo basin to the west, and the presence of three sea corridors in lower Central America to the north: the Atrato seaway, the Panama Portal, and the San Carlos Basin [72]. These marine transgressions resulted from changes in sea level that occurred during the warm climate periods of the Pliocene [73–75], but also during the Quaternary interglacial periods when warmer and wetter climates dominated again. Isolation during the cycles of sea incursions allowed rapid species evolution in the trans-Andean region, as has been recognized for several organisms by Nores [76], and is feasible that similar forces managed the divergence within *B*. *asper*.

Considering its South American origin, and given the low posterior probabilities for the ancestral locality that we retrieved for the root of Clade A (Fig 3), we postulated that the early separation between lineages in *B*. *asper* was driven by a dispersal event north into Caribbean Mesoamerica. In addition, the divergence between lineages in mid-Pliocene coincides with estimations of the final closure of Isthmian Central America, a dynamic event that, as previously mentioned, has shaped the region’s biogeography.

There is still considerable debate regarding the dynamics and timing of the final closure of Isthmian Central America. Some authors argue that the Isthmus arose as a series of islands in a shallow sea with a concluding land connection established in the late Pliocene [9, 10]; whereas others suggest that it emerged as a continuous land bridge since the Miocene [11, 72, 77], only broken by the three previously mentioned sea gates that eventually followed a north-to-south closure at the end of the Neogene [78]. Regardless of the paleogeographic model of emergence, colonization of Caribbean Mesoamerica by *B*. *asper* may have entailed crossing water corridors, a challenge that lancehead pitvipers seem suitable to carry out [65]. In support of this view, present-day surface currents in the southern Caribbean are mostly directed toward the northwest [79], and passive transportation from the north Caribbean coast of South America to northern Mesoamerica has been demonstrated experimentally [80].

According to our divergence time estimations, the separation within the South American clade B started in late Pliocene, when a group reached the Pacific coast of Isthmian Central America (PICA). The route involved in this expansion to Mesoamerica is unclear, but most likely it also involved passive transportation through the Atrato seaway when global climate was warm [81]. By then, the Talamanca mountain range in Isthmian Central America isolated this population from the Caribbean lowlands [82, 83]. This hypothesis explains why Caribbean and Pacific *B*. *asper* populations in Mesoamerica are not sister taxa. Close affinities between lineages along the Pacific slope of Isthmian Central America and those in the Choco region in Colombia have also been reported in other taxa: frogs [84, 85], birds [86] and snakes [23, 87], suggesting a comparable history of colonization.

Hence, we postulate that *B*. *asper* invaded Mesoamerica in at least two, chronologically separated independent events. This trend coincides with the two-step dispersal-pulse hypothesis proposed by Savage [88] to explain the unequal distribution of amphibians and reptiles of South American origin inhabiting Middle America. According to this author, the first and earlier episode took place some 3.4 MYA when the sea level lowered [89], and a handful of South American taxa were able to invade Mesoamerica before the final closure of the isthmus. Consequently, few South American taxa have distributions that extend as far north as Mexico. Savage’s [88] second dispersion episode presumably occurred almost a million years later, after the final closure of the Isthmian bridge. As an outcome of this late invasion, the majority of genera of South American origin have distributions that do not reach beyond southern Nicaragua or Costa Rica. Chronologically, Savage’s first pulse corresponds to the split of *B*. *asper* lineages, whereas his second pulse matches the invasion of *B*. *asper* from South America to PICA.

Alternatively, a single dispersal event into Middle America is also possible, especially if incomplete lineage sorting [90] or secondary (interspecific) gene flow is contemplated. Both are distinct phenomena but produce very similar patterns of shared genetic diversity that in turn could affect species integrity.

During range expansion, some alleles could increase their frequencies due to gene drift, thus, “surfing” into fixed spatial sectors at the expanding front. Lineages that arise as a consequence of demographic expansion are temporary but tend to last longer in species with limited dispersal abilities [91]. Genetic surfing has been shown to explain patterns of spatial assorting of gene variation in loci with weak effective dispersal, such as mitochondrial DNA. Thus, genetic surfing should be more frequent in species that exhibit male-biased dispersal, and that have relatively low vagility, as it has been recently reported in the coral snake *Micrurus tener* [92]. Although the dispersal patterns of *B*. *asper* are unknown, male-biased dispersal is expected in this species, as it has been described in several other species of vipers, comprising both crotalines [93] and viperines [94].

Our mismatch distribution analyses for demographic expansion provide evidence that MV and CCO groups represent recent populations that diverged in the early Pleistocene. Thus, it is possible that the sorting of these haplotype groups resulted from gene surfing and not due to divergence in allopatry. However, no further evidence for demographic expansion was recovered in other populations, not even in those located at the northern extreme of the species distribution. Furthermore, we did not observe divergent mitochondrial haplotypes in sympatry in the putative ancestral populations, which is a crucial component to evidence of gene surfing, therefore limiting our interpretation. Further sampling of these populations and the inclusion of genome-wide markers in the analyses could resolve the question of whether the spatial sorting of haplotype groups in *B*. *asper* resulted from allopatric divergence or genetic surfing during demographic expansion.

Another possible scenario is that mtDNA introgression from *B*. *atrox* into *B*. *asper* occurred in the past, as this could explain the paraphyletic relation of this last species retrieved in our analyses (Fig 2). Introgression between sibling species is more widespread than previously thought and is reported in a great variety of plant and animal taxa [95, 96] including snakes [97, 98]. Ancient introgression could occur even if no evidence of shared haplotypes is available, as has been recently reported by Ruane et al. [98] for milk snakes genus Lampropeltis. For species that exhibit male-biased dispersal, as expected in *B*. *asper*, rates of introgression in mtDNA markers are often higher compared with those from biparentally inhered nuclear DNA markers [95]. Unfortunately, we did not include nuclear markers in our analysis, and none of our mitochondrial haplotypes were shared among our phylogroups, thus precluding further comparisons to evaluate introgression.

### Relation between Caribbean Middle American and Ecuadorian groups

The observed sister relationship between MNCA/CICA and the WE/TS groups depicted in clade A (Fig 2) seems incongruous, as no biogeographical connection has ever been suggested between the mesic forests of Caribbean Middle American and the seasonally dry environments of southwestern Ecuador [99]. Biogeographically, the northern portion of Caribbean Middle America is dominated by Mesoamerican influences whereas southwestern Ecuador has influences from the Pacific dominion [100]. Therefore, biotic similarities of this last region are expected to be mainly with the geographically proximal Chocoan biogeographic region, as has been described for bats [101] and woody plants [102]. It is possible that observed affinities resulted from retention of ancestral polymorphisms followed by lineage sorting, but the sample size of our Ecuadorian populations prevent further analysis at this point.

### Phenotypic variation in *B*. *asper*

Across Mesoamerica, *B*. *asper* populations show remarkable differences in natural history traits [103], morphological variation [104, 105], venom composition [106, 107], and biological effects of venoms [108–110]. The extent of intraspecific variation in *B*. *asper* venoms could affect the capacity of antivenoms to neutralize toxins from snakes of geographically separated populations [111]; thus in Costa Rica polyvalent antivenom is produced from a mixture that includes B. asper venoms from the Caribbean and Pacific regions of that country [112, 113]. All these features suggest independence in the evolutionary history of at least the two *B*. *asper* lineages revealed in this study. In fact, Aragón and Gubensek [114] have suggested that given that the differences in morphology, toxin composition, and biological effect of venoms are so notable, populations in the Central American Isthmus warrant taxonomic recognition. Further, sequence divergences observed among *B*. *asper* Caribbean Mesoamerican populations and those in Pacific/South American (5.8 ± 0.2%) are on par to those reported among different species in other snake groups: *Lachesis stenophrys* and *L*. *melanocephala* with a 5.3% [115] the *Naja nigricollis* species complex between 4.7% to 8.3% [116], and the genus Agkistrodon between 4.0% to 6.4% [28].

Nevertheless, we refrain from proposing any taxonomic changes for these B. asper lineages at present, as their phylogenetic relationships with the *B*. *atrox* group remains unclear. Further phylogenetic resolution of our tree could be attained using other sequence markers, such as nuclear genes, as studies in birds [117], mammals [118, 119] and reptiles [120] suggest. These characters exhibit low homoplasy levels and are known to be useful in recovering phylogenetic information from taxonomic groups that have experienced adaptive radiation in a short period [119], therefore resulting in more robust phylogenies. Also, a more thorough sampling that includes other localities from Panama and especially from Venezuela and Ecuador might be necessary to further resolve the phylogenetic affinities within the group.

### Concluding remarks

Divergence within *B*. *asper* lineages appears to result from the tectonic events and sea transgressions that have shaped Mesoamerica and northern South America since the early Pliocene. Our findings support the view that *B*. *asper* invaded Mesoamerica multiple times during the complex history of the final closure of Isthmian portal, and that Savage’s [88] view of two invasion pulses to explain differential distribution of taxa of South American origin in Mesoamerica might hold for this species. However, our data suggest that *Bothrops asper*, as currently understood, probably consists of a complex of related lineages that follow their evolutionary trajectories. A more robust revision of these lineages and their taxonomic status is presently underway.

## Supporting information

S1 Table Specimens examined. Accession Numbers With Asterisks Are Sequences Obtained Of GeneBank.
(DOCX)
