# Supplementary material for: Phylogeography of the Central American lancehead Bothrops asper (SERPENTES: VIPERIDAE)
Source: PLoS One. 2017 Nov 27;12(11):e0187969. doi: 10.1371/journal.pone.0187969 (PMC5703453; doi:10.1371/journal.pone.0187969)
Supplement: S1 Table — Accession Numbers With Asterisks Are Sequences Obtained Of GeneBank. (DOCX) [file pone.0187969.s001.docx]

**S1 Table.** Specimens Examined. Accession numbers with asterisks are sequences obtained from GeneBank. Group refers to *B. asper* phylogroups described in text.

| **Species** | **Collection locality** | **Country** | **Voucher No.** | **Group** | **Accession No.** | |
| --- | --- | --- | --- | --- | --- | --- |
|  |  |  |  |  | **Cytb** | **ND4** |
| *Agkistrodon contortrix* | Athens Co, Ohio | USA | MOODY 338 |  | AY223612* | AF157576* |
| *Agkistrodon piscivorous* | South Carolina | USA | CLP 30 |  | AY223615* | AF156578* |
| *Bothriopsis bilineata* | Macuma, Morona Santiago | Ecuador | FHGO 982 |  | AF292592* | AF292630* |
| *Bothriopsis chromelas* | Pasco Dept. | Peru | LSUMZ 41037 |  | DQ305471* | DQ305488* |
| *Bothriopsis taeniata* |  | Ecuador | FHG 195 |  | AF292591* | AF292629* |
| *Bothrocophias campbelli* | Chimborazo, Pallatanga | Ecuador | INHMT |  | AF191582* | AF292622* |
| *Bothrocophias hyoprora* | Leticia | Colombia |  |  | AY223593* | U41886* |
| *Bothrocophias microphthalmus* |  | Ecuador | FHGO 2566 |  | AF292577* | AF292615* |
| *Bothropoides alcatraz* | Ilha de Alcatrazes | Brazil | CBGM-baz005 |  | AY865824* |  |
| *Bothropoides diporus* | La Rioja | Argentina | PT3404 |  | DQ305472* | DQ305489* |
| *Bothropoides erythromelas* | Algóas | Brazil | RG-829 |  | AY223600* | U41877* |
| *Bothropoides insularis* | São Paulo, Ilha da Queimada Grande | Brazil |  |  | AF292590* | AF292628* |
| *Bothropoides jararaca* |  | Brazil |  |  | AY865819* |  |
| *Bothropoides lutzi* | Uruçuí-Una | Brazil | MZUSP12536 |  | KF801136* | KF801264* |
| *Bothropoides marmoratus* | Goiânia | Brazil | CEPB8171 |  | KF801137* | KF801265* |
| *Bothropoides mattogrossensis* | Cuiabá | Brazil | NORMAT109 |  | KF801151 * | KF801279* |
| *Bothropoides neuwiedi* | Votorantim | Brazil | BSP73637 |  | KF801201* |  |
| *Bothropoides pauloensis* | Analândia | Brazil | IBSP71785 |  | KF801202* | KF801320* |
| *Bothropoides pubescens* | Barão do Triunfo | Brazil | NOPA3860 |  | KF801227 | KF801344* |
| *Bothrops asper* | Mile 38, Western Highway, Belize Zoo | Belize | WW 264 | NCA | A292600* | AF292638* |
| *Bothrops asper* | Mile 16.5, Sand Hill, Belize Dist | Belize | WW 252 | NCA | MG265759 | MG265867 |
| *Bothrops asper* | Rancho Dolores, 32.7 km Hill Bank | Belize | WW 263 | NCA | MG265760 | MG265868 |
| *Bothrops asper* | Btw. Burrell Boom and Hattieville, | Belize | WW 271 | NCA | MG265762 | MG265870 |
| *Bothrops asper* | 10.7 km from Hill Bank, Orange Walk | Belize | WW 270 | NCA | MG265761 | MG265869 |
| *Bothrops asper* | Bahía Solano , Choco | Colombia | SUA 1223 | CHOCO 1 | MG265746 | MG265857 |
| *Bothrops asper* | Posamanza, Choco | Colombia | SUA 1576 | CHOCO 1 | MG265745 | MG265856 |
| *Bothrops asper* | Buenaventura, Valle del Cauca | Colombia | INS B60 | CHOCO 1 | FJ985702* | FJ985713* |
| *Bothrops asper* | Carepa, Antioquia | Colombia | SUA 3241 | CHOCO 1 | MG265749 | MG265859 |
| *Bothrops asper* | Lloró , Chocó | Colombia | MSC 1 | CHOCO 1 | MG265747 | MG265858 |
| *Bothrops asper* | Lloró, Chocó | Colombia | SUA 3818 | CHOCO 1 | MG265748 |  |
| *Bothrops asper* | Mutatá, Antioquia | Colombia | SUA 3487 | CHOCO 1 | MG265751 | MG265861 |
| *Bothrops asper* | Nuquí, Choco | Colombia | MHUA 14438 | CHOCO 1 | FJ985703* | FJ985714* |
| *Bothrops asper* | Nuquí, Choco | Colombia | MHUA 14437 | CHOCO 1 | MG265750 | MG265860 |
| *Bothrops asper* | Unguía, Choco | Colombia | SUA 1280 | CHOCO 1 | MG265744 | MG265855 |
| *Bothrops asper* | Cáceres, Antioquia | Colombia | SUA 2256 | CCO | MG265739 | MG265850 |
| *Bothrops asper* | Caucasia, Antioquia | Colombia | SUA 2698 | CCO | MG265740 | MG265851 |
| *Bothrops asper* | Caucasia, Antioquia | Colombia | SUA 2375 | CCO | MG265740 | MG265852 |
| *Bothrops asper* | Caucasia, Antioquia | Colombia | MHUA 14447 | CCO | FJ985699* | FJ985711* |
| *Bothrops asper* | Fundación, Magdalena | Colombia | SUA 3971 | CCO | FJ985700* | FJ985712* |
| *Bothrops asper* | Necoclí, Antioquia | Colombia | SUA 2937 | CCO | MG265738 | MG265849 |
| *Bothrops asper* | Ocaña, Norte de Santander | Colombia | SUA 3395 | CCO | MG265737 | MG265848 |
| *Bothrops asper* | Planeta Rica, Córdoba | Colombia | SUA 2909 | CCO | MG265743 | MG265854 |
| *Bothrops asper* | Puerto Valdivia, Antioquia | Colombia | SUA 2696 | CCO | MG265742 | MG265853 |
| *Bothrops asper* | Tierralta, Cordoba | Colombia | MHUA 14327 | CCO | MG265736 | MG265847 |
| *Bothrops asper* | Turbaco, Bolívar | Colombia | SUA 3850 | CCO | MG265735 | MG265846 |
| *Bothrops asper* | Turbaco, Bolívar | Colombia | SUA 3849 | CCO | MG265734 |  |
| *Bothrops asper* | Cimitarra, Santander | Colombia | SUA 1691 | MV | FJ985698* | FJ985710* |
| *Bothrops asper* | Cimitarra, Santander | Colombia | SUA 2388 | MV | MG265723 | MG265837 |
| *Bothrops asper* | Cimitarra, Santander | Colombia | SUA 2446 | MV | MG265724 | MG265838 |
| *Bothrops asper* | Cimitarra, Santander | Colombia | SUA 2464 | MV | MG265726 | MG265839 |
| *Bothrops asper* | Gómez Plata, Antioquia | Colombia | MHUA 14400 | MV | MG265727 | MG265840 |
| *Bothrops asper* | Ibagué, Tolima | Colombia | SUA 2990 | MV | MG265730 | MG265842 |
| *Bothrops asper* | La Victoria, Caldas | Colombia | MHUA 14444 | MV | MG265719 | MG265834 |
| *Bothrops asper* | Maceo, Antioquia | Colombia | SUA 3124 | MV | MG265714 | MG265832 |
| *Bothrops asper* | Maceo, Antioquia | Colombia | SUA 3123 | MV | MG265713 | MG265831 |
| *Bothrops asper* | Maceo, Antioquia | Colombia | MHUA 14310 | MV | MG265712 | MG265830 |
| *Bothrops asper* | Maceo, Antioquia | Colombia | MHUA14311 | MV | FJ985697* | FJ985709* |
| *Bothrops asper* | Maceo, Antioquia | Colombia | SUA 2972 | MV |  | MG265828 |
| *Bothrops asper* | Mariquita, Tolima | Colombia | SUA 3961 | MV | MG265715 | MG265833 |
| *Bothrops asper* | Porce, Antioquia | Colombia | SUA 3367 | MV | MG265721 | MG265836 |
| *Bothrops asper* | Puerto Berrío, Antioquia | Colombia | SUA 2119 | MV | MG265709 | MG265819 |
| *Bothrops asper* | Puerto Berrío, Antioquia | Colombia | SUA 2311 | MV |  | MG265898 |
| *Bothrops asper* | Puerto Berrío, Antioquia | Colombia | SUA 3375 | MV |  | MG265827 |
| *Bothrops asper* | Puerto Berrío, Antioquia | Colombia | SUA 2243 | MV |  | MG265823 |
| *Bothrops asper* | Puerto Berrío, Antioquia | Colombia | SUA 1284 | MV |  | MG265822 |
| *Bothrops asper* | Puerto Berrío, Antioquia | Colombia | SUA 2721 | MV | FJ985696* | FJ985708* |
| *Bothrops asper* | Puerto Berrío, Antioquia | Colombia | SUA 2720 | MV | MG265711 | MG265821 |
| *Bothrops asper* | Puerto Berrío, Antioquia | Colombia | SUA 2683 | MV | MG265710 | MG265820 |
| *Bothrops asper* | Río Claro, Antioquia | Colombia | MSC 2 | MV | MG265731 | MG265843 |
| *Bothrops asper* | San Carlos, Antioquia | Colombia | SUA 2978 | MV | MG265732 | MG265844 |
| *Bothrops asper* | San Carlos, Antioquia | Colombia | SUA 2344 | MV | MG265722 |  |
| *Bothrops asper* | San Carlos, Antioquia | Colombia | SUA 2043 | MV | MG265718 |  |
| *Bothrops asper* | San Luis, Antioquia | Colombia | SUA 3174 | MV | MG265717 |  |
| *Bothrops asper* | San Luis , Antioquia | Colombia | SUA 2783 | MV | MG265716 |  |
| *Bothrops asper* | San Miguel, Antioquia | Colombia | SUA 3783 | MV | MG265728 | MG265841 |
| *Bothrops asper* | San Rafael, Antioquia | Colombia | SUA 3333 | MV | MG265733 | MG265845 |
| *Bothrops asper* | Sonsón, Antioquia | Colombia | SUA 2782 | MV |  | MG265829 |
| *Bothrops asper* | Vegachí, Antioquia | Colombia | SUA 3346 | MV |  | MG265826 |
| *Bothrops asper* | Vegachí, Antioquia | Colombia | SUA 2784 | MV |  | MG265825 |
| *Bothrops asper* | Vegachí, Antioquia | Colombia | SUA 2732 | MV |  | MG265824 |
| *Bothrops asper* | Yolombó, Antioquia | Colombia | SUA 3050 | MV | MG265720 | MG265835 |
| *Bothrops asper* | Yondó, Antioquia | Colombia | SUA 2707 | MV | MG265729 |  |
| *Bothrops asper* | Sarapiqui, Heredia | Costa Rica | DPL 2900 | CICA | MG265754 | MG265862 |
| *Bothrops asper* | Peñas Blancas | Costa Rica | MSC 3 | CICA | MG265756 | MG265864 |
| *Bothrops asper* | Laguna Zarcero, Alajuela | Costa Rica | WW 1320 | CICA | MG265770 | MG265876 |
| *Bothrops asper* | Siquirres, Limón | Costa Rica | WW 1318 | CICA | MG265771 | MG265877 |
| *Bothrops asper* | San Carlos, Alajuela | Costa Rica | ICP 405 | CICA | MG265753 |  |
| *Bothrops asper* | San Carlos, Alajuela | Costa Rica | ICP Atl 04-04 | CICA | FJ985706* | FJ985716* |
| *Bothrops asper* | San Carlos, Alajuela | Costa Rica | ICP Atl 03-01 | CICA | MG265755 | MG265863 |
| *Bothrops asper* | Quebrada Ganado, San José | Costa Rica | MZUCR-11152 | PICA | AY223599* | U41876 * |
| *Bothrops asper* | Acosta , San José | Costa Rica | ICP 461 | PICA | MG265752 |  |
| *Bothrops asper* | Acosta , San José | Costa Rica | ICP Pac 02-02 | PICA | FJ985704* |  |
| *Bothrops asper* | Quebrada La Gamba, Puntarenas | Costa Rica | MSC 4 | PICA | MG265781 | MG265887 |
| *Bothrops asper* | Carretera Interamericana, Puntarenas | Costa Rica | MSC 5 | PICA | MG265782 | MG265888 |
| *Bothrops asper* | Manantial de Colonche, Guayas | Ecuador | FHGO 1564 | WE | AF292601* | AF292639* |
| *Bothrops asper* | Vilcabamba, Loja | Ecuador | WW 740 | TR | MG265775 | MG265881 |
| *Bothrops asper* | Vilcabamba, Loja | Ecuador | FHGO 1263 | TR | MG265776 | MG265882 |
| *Bothrops asper* | Pedro Vicente Maldonado, Pichincha | Ecuador | FHGO 2199 | CHOCO 2 | MG265773 | MG265879 |
| *Bothrops asper* | Pedro Vicente Maldonado, Pichincha | Ecuador | FHGO 2167 | CHOCO 2 | MG265772 | MG265878 |
| *Bothrops asper* | Sacramento, Pallatanga, Chimborazo | Ecuador | WW-INHMT 1 coll | WE | MG265777 | MG265883 |
| *Bothrops asper* | Sacramento, Pallatanga, Chimborazo | Ecuador | WW-INHMT 3 coll | WE | MG265779 | MG265885 |
| *Bothrops asper* | Flavio Alfaro, Manabí | Ecuador | WW-INHMT 4 coll | WE | MG265780 | MG265886 |
| *Bothrops asper* | Vinces, Los Rios | Ecuador | WW-INHMT 2 coll | WE | MG265778 | MG265884 |
| *Bothrops asper* | Nanegalito, Pichincha | Ecuador | FHGO 1099 | CHOCO 2 | MG265774 | MG265880 |
| *Bothrops asper* | Quebrada El Branchi, Izabal | Guatemala | ENS 6062 | NCA | MG265757 | MG265865 |
| *Bothrops asper* | Gringo Perdido, Lago Petén Itzá | Guatemala | JAC 21101 | NCA | MG265758 | MG265866 |
| *Bothrops asper* | Peten | Guatemala | ICP 1150 | MY | FJ985707* |  |
| *Bothrops asper* | Copan | Honduras | ICP 1282 | NCA | MG265769 | MG265875 |
| *Bothrops asper* | Sierra de los Tuxtlas, Veracruz | Mexico | ENS 10377 | MY | MG265766 | MG265872 |
| *Bothrops asper* | Los Tuxtlas, Veracruz | Mexico | WW 875 | MY | MG265767 | MG265873 |
| *Bothrops asper* | Los Tuxtlas, Veracruz | Mexico | WW 876 | MY | MG265768 | MG265874 |
| *Bothrops asper* | Municipio Zongolica: Aticpac, Veracruz | Mexico | JAC 22893 | MY | MG265765 |  |
| *Bothrops asper* | San Martin, Veracruz | Mexico | JRM 4044 | MY | MG265764 |  |
| *Bothrops asper* | Carretera Tuxtepec, Oaxaca | Mexico | ENS 9995 | MY | MG265763 | MG265871 |
| *Bothrops asper* | Matagalpa | Nicaragua | ENS 9834 | CICA | FJ985705* |  |
| *Bothrops asper* | La Changa, Darién | Panama | ICP1286 | CHOCO 1 | FJ985701* | FJ985715* |
| *Bothrops asper* | La Tronosa, Los Santos | Panama | MSM 17 | PICA | MG265783 | MG265889 |
| *Bothrops asper* | Cocoli, Panama | Panama | MSM 73 | PICA | MG265786 | MG265891 |
| *Bothrops asper* | Cerro Azul, Panama City | Panama | MSM 263 | PICA | MG265788 | MG265892 |
| *Bothrops asper* | San Andres, Chiriqui | Panama | MSM 27 | PICA | MG265784 | MG265890 |
| *Bothrops asper* | Capira, Altos de María, Panama | Panama | MSM 266 | PICA | MG265789 | MG265893 |
| *Bothrops asper* | Chiriqui | Panama | MSM J4 | PICA | MG265794 | MG265897 |
| *Bothrops asper* | Chiriqui | Panama | MSM J1 | PICA | MG265792 | MG265895 |
| *Bothrops asper* | Chiriqui | Panama | MSM J3 | PICA | MG265793 | MG265896 |
| *Bothrops asper* | Quebrada Cascajal, Cocle | Panama | MSM 241 | CHOCO 1 | MG265787 |  |
| *Bothrops asper* | Cocoli | Panama | MSM 72 | CHOCO 1 | MG265785 |  |
| *Bothrops asper* | Canal Zone | Panama | UXO | CHOCO 1 | MG265725 |  |
| *Bothrops asper* | Cocoli, Panama | Panama | MSM 299 | CHOCO 1 | MG265790 | MG265894 |
| *Bothrops asper* | Parque Soberania, Panama | Panama | MSM 254 | CHOCO 1 | MG265791 |  |
| *Bothrops atrox* | Meta | Colombia | SUA 2198 |  | MG265700 | MG265804 |
| *Bothrops atrox* | Fomeque, Cundinamarca | Colombia | INS B01 |  | MG265701 |  |
| *Bothrops atrox* | Agua | Ecuador |  |  | MG265702 |  |
| *Bothrops atrox* | Pucallpa | Perú |  |  | MG265703 |  |
| *Bothrops atrox* | Diego Martin, via Garth Underwood | Trinidad | 196 coll. Hans B. |  | MG265704 | MG265805 |
| *Bothrops atrox* |  |  | WWW-743 |  | AY223598* | AY223641* |
| *Bothrops brazili* | Morona Santiago, Macuma | Ecuador | FHGO 982 |  | AF292597* | AF292635* |
| *Bothrops caribbaeus* | Grande Anse | Saint Lucia |  |  | AF292598* | AF292636* |
| *Bothrops colombiensis* | Altagracia de Orituco, Guatopo, E. Miranda | Venezuela | WW 73 |  | MG265795 | MG265817 |
| *Bothrops colombiensis* | Altagracia de Orituco, Guatopo ,E. Guarico | Venezuela | WW 74 |  | AF292602* | AF292640* |
| *Bothrops isabelae* | Pajarito , Boyacá | Colombia | INS B32 |  | MG265705 | MG265806 |
| *Bothrops isabelae* | Arauca | Colombia | INS B22 |  | MG265706 | MG265807 |
| *Bothrops isabelae* | Nr. Guaibacoa, Falcon | Venezuela | WW 778 |  | MG265800 | MG265812 |
| *Bothrops isabelae* | Santa Cruz de Bucaral, Falcon | Venezuela | WW 777 |  | MG265796 | MG265808 |
| *Bothrops isabelae* | Cabure, Falcon | Venezuela | WW 783 |  | MG265797 | MG265809 |
| *Bothrops isabelae* | San Francisco, Falcon | Venezuela | WW 785 |  | MG265798 | MG265810 |
| *Bothrops isabelae* | S Curarima, Falcon | Venezuela | WW 776 |  | MG265799 | MG265811 |
| *Bothrops isabelae* | P.N. Guanche, nr. Ospino, Portuguesa | Venezuela | WW 764 |  | MG265801 | MG265813 |
| *Bothrops isabelae* | P.N. Guanche, nr. Ospino, Portuguesa | Venezuela | WW 763 |  | MG265802 | MG265814 |
| *Bothrops isabelae* | Guanare, Portuguesa | Venezuela | WW 79 |  | AF292603* | AF292641* |
| *Bothrops isabelae* | Caño Delgadito Rd., Portuguesa | Venezuela | WW 773 |  | MG265708 | MG265816 |
| *Bothrops isabelae* | Caño Delgadito Rd. Portuguesa | Venezuela | WW 772 |  | MG265707 | MG265815 |
| *Bothrops jararacussu* | São Paulo: Cananéia | Brazil | IB 55313 |  | AF292596* | AF292634* |
| *Bothrops lanceolatus* |  | Martinique |  |  | AF292599* | AF292637* |
| *Bothrops leucurus* | Porto Seguro, Bahía | Brazil | IB 55480 |  | AF246273* | AF246284* |
| *Bothrops marajoensis* | Para , Ilha de Marajo | Brazil |  |  | AF292605* | AF292643* |
| *Bothrops moojeni* | Brasilia | Brazil | IB 56558 |  | AF292606* | AF292644* |
| *Bothrops osbornei* | Pichincha, Pedro Vicente | Ecuador | FHGO live 2166 |  | AF292595* | AF292633* |
| *Bothrops punctatus* |  |  | FHGO live 2452 |  | AF292594* | AF292632* |
| *Bothrops venezuelensis* |  | Venezuela | WW 1145 |  | MG265698 |  |
| *Bothrops venezuelensis* |  | Venezuela | WW 1147 |  | MG265699 |  |
| *Bothrops venezuelensis* | El Ávila or Rancho Grande | Venezuela | WW 1144 |  | MG265697 | MG265803 |
| *Crotalus atrox* | Texas | USA | CLP 64 |  | AY223608* | AY223646* |
| *Crotalus ruber* |  | USA | RWV 2001-08 |  |  | DQ679838* |
| *Lachesis muta* |  | Perú | Cadle 135 |  | AY223604* | AY223644 |
| *Lachesis stenophrys* | Limón | Costa Rica |  |  | AY223603* | U41885 |
| *Ophryacus melanurus* |  | México | UTA-R34605 |  | AY223587* | AY223634* |
| *Ophryacus undulatus* |  | México |  |  | AY223586* | AY223633* |
| *Rhinocerophis alternatus* |  |  | DLP 2879 |  | AY223601* | AY223642* |
| *Rhinocerophis ammodytoides* | Neuguen | Argentina | MVZ 223514 |  | AY223595* | AY223639* |
| *Rhinocerophis cotiara* | Santa Catarina | Brazil | IB Live Col 3829 |  | AF292581* | AF292619* |
| *Rhinocerophis fonsecai* | São Paulo, Campos do Jordão | Brazil | IB55543 |  | AF292580* | AF292618* |
| *Sistrurus catenatus* | Texas | USA | MOODY 502 |  | AY223610* | AY223648* |
